# Characterization of vitamin D supplementation and clinical outcomes in a large cohort of early Parkinson’s disease

**DOI:** 10.1186/s40734-018-0074-6

**Published:** 2018-10-31

**Authors:** Nijee S. Luthra, Soeun Kim, Yunxi Zhang, Chadwick W. Christine, On behalf of the NINDS NET-PD Investigators

**Affiliations:** 1 Department of Neurology, University of California, 1635 Divisadero, Suite 520-530, San Francisco, CA 94115 USA; 2 Department of Biostatistics and Data Science, University of Texas Health Center, 1200 Pressler Street, Houston, TX 77030 USA; 3 Department of Neurology, University of California San Francisco, 400 Parnassus Ave Box 0348, San Francisco, CA 94122 USA

**Keywords:** 1,25-dihydroxyvitamin D, Parkinson’s disease

## Abstract

**Background:**

Vitamin D (VitD) deficiency is common in Parkinson’s disease (PD) and has been raised as a possible PD risk factor. In the past decade, VitD supplementation for potential prevention of age related conditions has become more common. In this study, we sought to characterize VitD supplementation in early PD and determine as an exploratory analysis whether baseline characteristics or disease progression differed according to reported VitD use.

**Methods:**

We analyzed data from the National Institutes of Health Exploratory Trials in Parkinson’s Disease (NET-PD) Long-term study (LS-1), a longitudinal study of 1741 participants. Subjects were divided into following supplement groups according to subject exposure (6 months prior to baseline and during the study): no VitD supplement, multivitamin (MVI), VitD ≥400 IU/day, and VitD + multivitamin (VitD+MVI). Clinical status was followed using the Unified Parkinson’s Disease Rating Scale, Symbol Digit Modalities Test, total daily levodopa equivalent dose, and Parkinson’s Disease Questionnaire.

**Results:**

About 5% of subjects took VitD alone, 7% took VitD+MVI, 34% took MVI alone, while 54% took no supplement. Clinical outcomes at 3 years were similar across all groups.

**Conclusion:**

This study shows VitD supplementation ≥400 IU/day was not common in early PD and that its use was similar to that seen in the US population. At 3 years, there was no difference in disease progression according to vitamin D supplement use.

## Background

Numerous reports have shown that vitamin D (VitD) insufficiency is common in aging, is associated with greater risk of falls and fractures, and that VitD supplementation may reduce the risk of fractures [1]. Because of this observation, and possibly because of numerous studies showing associations of D deficiency with diseases in adults, VitD supplementation has become common. A cross-sectional survey from 2011 to 12 found that about 21% of adults aged 40–64 and 38% of those > 65 take a separate VitD supplement [2].

A relationship of VitD levels and Parkinson’s disease (PD) was first recognized in 1997 when Sato and colleagues reported a higher prevalence of VitD deficiency and reduced bone mass in PD [3]. Since then, a number of studies have confirmed these findings [4] and that a mutation in the VitD receptor is associated with an increased risk of developing PD [5]. Studies in experimental animal models of PD have demonstrated neuroprotective effects of VitD [6], lending support to the hypothesis that VitD status may influence PD pathogenesis. The Mini-Finland Health Survey provided additional support of this hypothesis by showing a greater risk for PD in those with lower VitD levels [7].

Because these studies raise the question as to whether VitD supplementation affects PD progression, we performed a study to characterize contemporary VitD supplementation in a large, longitudinal cohort of early PD patients from the National Institutes of Health Exploratory Trials in Parkinson’s Disease (NET-PD) Long-term study (LS-1) which enrolled subjects from the United States and Canada from 2007 to 2010. As a an exploratory outcome, we also determined whether baseline characteristics or disease progression outcomes differed according to reported VitD supplement use prior to baseline and during the study observation period.

## Methods

The NET-PD LS-1 study was a large randomized multicenter, double-blind, placebo-controlled study of creatine monohydrate. As previously described, eligible subjects had diagnosis of PD for < 5 years and had started treatment with levodopa or dopamine agonist for ≥90 days but < 2 years prior to the baseline visit [8]. Subjects were monitored with annual visits for up to 6 years. The study was halted before completion after it was determined no difference would emerge between the treatment arms [8].

At study entry, subjects were required to report all medications and supplements used in the prior 180 days, although many provided much longer histories of supplement use. We reviewed the concomitant medication records for VitD and multivitamin (MVI) use including supplement brand name (when available), dose, frequency, start date and end date. Of the 1956 records of supplement use (separate records were created for each supplement), 620 (31%) documented supplement use starting prior to 2005. For those taking specified supplements, the published dose was used. The daily dose was calculated according to dose and frequency. Subjects were stratified according to the following VitD exposure groups: no VitD or MVI supplement (NoSupp), multivitamin where dose of VitD was assumed (if not specified) to be 400 IU/dose (MVI group), ≥ 400 IU/day of VitD (VitD group), or multivitamin plus ≥ of 400 IU/day of VitD (VitD+MVI group).

In order to construct defined groups of participants with different dose and longitudinal VitD exposures, subjects were included in the analysis if they had been taking supplements for ≥90 days prior to baseline and continued that supplement for at least 1.5 years of the first 3 years of the study or their records indicated no D supplementation or MVI during this timeframe (NoSupp group). Subjects who took VitD for shorter durations during the study period or at lower doses were excluded.

### Assessments

Because a significant fraction of subjects did not complete the 5-year visit due to early study termination, we calculated mean study outcomes at baseline and the change from baseline in outcomes at 3 years. Outcomes included demographics, Unified Parkinson’s Disease Rating Scale (UPDRS) total score and its subscores obtained in the practically defined “On-medication” state, [mental (Part 1), activities of daily living (Part 2), and motor (Part 3)], Parkinson’s Disease Questionnaire (PDQ39), Scales for Outcomes in Parkinson’s Disease-Cognition (SCOPA-COG; only performed at baseline and 5 year visit), Symbol Digit Modalities Test (SDMT), and total daily levodopa equivalent dose (LED).

### Statistical analysis

To investigate baseline characteristics for each group, means and standard deviations were calculated for continuous variables, and percentages were computed for categorical variables. NoSupp, MVI, VitD, VitD+MVI groups were compared using Analysis of variance (ANOVA) test for continuous variables, and Chi-square tests and Fisher’s exact tests for categorical variables. Baseline age and age at PD diagnosis was summarized by performing subgroup analyses on males and females, using ANOVA test within each gender group. Pairwise comparisons were assessed using the Hochberg’s multiple testing method. Changes from baseline in the outcomes were computed at the third year visit, and mean changes were compared for four groups using ANOVA test. The ANOVA test was used to compare mean age at diagnosis for the four supplement groups, within each age categories. In order to adjust for baseline morbidity, we also performed the outcome analysis using mixed models, adjusting for baseline age and the corresponding baseline score as fixed effects and including site as a random effect. SAS 9.4 was used for the analyses.

## Results

Of the 1741 subjects who enrolled in the study, 943 subjects had been taking MVI, VitD, or VitD+MVI for ≥90 days prior to baseline and at least 1.5 years of duration of the study or reported no supplement use (NoSupp group) during this timeframe. Of these subjects, 54% of subjects took NoSupp, 34% took MVI, 5% took VitD, and 7% took VitD+MVI (where for VitD, the dose was > 400 IU/day).

Although disease severity as measured by rating scales was similar between the groups at baseline, the age at baseline, and the related age at diagnosis of PD, was higher in subjects taking supplements [ MVI (60.2 ± 8.7), VitD (63.9 ± 6.6) and VitD+MVI (64.1 ± 7.7)] compared to subjects in NoSupp (58.5 ± 9.7) group (Table 1). In order to address whether supplement use differed according to age, we then performed an analysis segregated according to age range (< 50, 50-59, 60–69, ≥70) at PD diagnosis (Table 2). This analysis confirmed that VitD use was higher in older patients and that when sorted by age range, the mean ages at diagnosis were not different. Time from diagnosis to start of dopaminergic treatment was not different in subjects taking supplements (Table 1). The percentage of females and non-Hispanic whites was higher in the VitD and VitD+MVI groups.

**Table 1:** Baseline Characteristics

	No Supplement (*N* = 511)	MVI	D	D + MVI	*P* value
		(*N* = 322)	(*N* = 42)	(*N* = 68)	
	Mean (SD)				F test
Age in years	60.1 (9.7)	61.8 (8.6)^*^	65.3 (6.4)^*^	65.5 (7.7)^*^	< 0.0001
Age at PD Dx	58.5 (9.7)	60.2 (8.7)^*^	63.9 (6.6)^*^	64.1 (7.7)^*^	< 0.0001
Yrs since Dx	1.6 (1.1)	1.7 (1.2)	1.3 (1.0)	1.4 (0.8)	0.07
Years since Dx to D-treatment	1.1 (1.1)	1.3 (1.2)	0.9 (1.0)	1.0 (0.8)	0.08
UPDRS total	25.1 (10.3)	25.4 (10.3)	25.1 (11.4)	24.5 (9.8)	0.92
UPDRS mental	1.2 (1.3)	1.2 (1.3)	1.4 (1.4)	1.2 (1.3)	0.85
UPDRS ADL	6.8 (3.7)	6.8 (3.6)	6.2 (3.9)	6.4 (3.4)	0.66
PDQ 39 summary	12.9 (10.4)	11.5 (8.7)	12.5 (9.6)	10.5 (8.3)	0.08
SCOPA COG	30.4 (5.1)	30.4 (5.2)	30.9 (4.6)	31.1 (5.0)	0.71
SDMT	45.1 (11.1)	44.4 (10.9)	45.2 (11.4)	45.9 (11.4)	0.71
Total daily LED	374.9 (224.9)	371.3 (232.8)	390.0 (208.0)	367.4 (355.4)	0.96
	Frequency (%)				X^2^test^a^
Female %	144 (28.2%)	94 (29.2%)	27 (64.3%)	38 (55.9%)	< 0.0001
Non-Hispanic white	448 (87.7%)	303 (94.1%)	41 (97.6%)	66 (97.1%)	0.001
Education > 17 years^a^	149 (29.2%)	118 (36.7%)	16 (38.1%)	18 (26.5%)	0.08

*Abbreviations*: *PD* Parkinson’s disease, *Dx* diagnosis, *yrs* years, *D-treatment* dopaminergic treatment (dopamine agonist or levodopa), *UPDRS* Unified Parkinson’s Disease Rating Scale, *ADL* activities of daily living, *PDQ* Parkinson’s disease questionnaire, *SCOPA COG* Scales for outcomes of Parkinson’s disease-Cognition, *SDMT* symbol digit modalities test, *LED* levodopa equivalent dose

^*^p < .05 compared to NoSupp, according to pairwise comparisons Hochberg method

^a^X^2^ test was calculated by Fisher’s exact test

**Table 2:** Analysis of Supplement Use by Age Range

Age at Diagnosis	Statistics	No Supp (*N* = 511)	MVI (*N* = 322)	VitD (*N* = 42)	VitD + MVI (*N* = 68)	*p*-value
< 50	Freq (%)	91 (70%)	35 (27%)	1 (1%)	2 (2%)	
	Mean (SD)	44.04 (4.54)	44.63 (5.07)	45.00 (NA)	46.50 (3.54)	0.83
50–59	Freq (%)	182 (58%)	106 (34%)	10 (3%)	17 (5%)	
	Mean (SD)	54.85 (2.73)	54.87 (2.96)	56.70 (3.09)	56.06 (2.88)	0.08
60–69	Freq (%)	164 (46%)	139 (39%)	23 (6%)	30 (9%)	
	Mean (SD)	63.81 (2.90)	64.12 (2.85)	64.83 (3.16)	64.13 (2.76)	0.42
≥70	Freq (%)	74 (52%)	42 (29%)	8 (6%)	19 (13%)	
	Mean (SD)	73.54 (3.21)	73.29 (2.62)	72.38 (1.06)	73.37 (3.92)	0.78

No differences in mean clinical outcomes were noted between groups at 3 years (Table 3). An analysis using mixed models, adjusted for baseline age and corresponding baseline scores as covariates, also failed to show a difference in outcomes according to supplement use (Table 4).

**Table 3:** Mean Change in Outcomes and at 3-year Visit

	No D Supplement (N = 511)	MVI (*N* = 322)	D (*N* = 42)	D + MVI (*N* = 68)	*P* value
	Mean (SD)				F test
Change in UPDRS total	5.5 (11.8)	6.2 (12.0)	4.9 (9.8)	9.2 (14.3)	0.12
Change in UPDRS mental	0.5 (1.5)	0.5 (1.5)	0.6 (2.0)	0.6 (1.8)	0.83
Change in UPDRS ADL	2.0 (4.4)	2.3 (4.5)	1.9 (3.6)	3.0 (4.7)	0.30
Change in PDQ 39 summary	4.6 (10.4)	4.3 (9.6)	4.0 (11.3)	4.8 (10.5)	0.96
Change in SDMT	0.002 (9.5)	−0.2 (9.4)	−1.6 (11.0)	−0.8 (13.3)	0.73
Change in Total daily LED	294.6 (367.1)	268.4 (339.8)	220.6 (244.5)	271.5 (346.4)	0.48

*Abbreviations*: *UPDRS* Unified Parkinson’s Disease Rating Scale, *ADL* activities of daily living, *PDQ* Parkinson’s disease questionnaire, *SDMT* symbol digit modalities test, *LED* levodopa equivalent dose

**Table 4:** Change in Outcomes at 3-year Visit according to mixed models^a^

Outcome	Group	Estimate	*P* value
Change in UPDRS total	No Supplement	.	.
	MVI	0.248	0.77
	D	−1.517	0.43
	D + MVI	2.540	0.10
Change in UPDRS ADL	No Supplement	.	.
	MVI	0.117	0.70
	D	−0.555	0.43
	D + MVI	0.469	0.41
Change in UPDRS mental	No Supplement	.	.
	MVI	−0.058	0.58
	D	0.083	0.73
	D + MVI	0.083	0.67
Change in PDQ39	No Supplement	.	.
	MVI	−0.571	0.43
	D	− 0.986	0.55
	D + MVI	−0.422	0.75
Change in SDMT	No Supplement	.	.
	MVI	−0.041	0.95
	D	−0.654	0.67
	D + MVI	0.871	0.48
Change in Total daily LED	No Supplement	.	.
	MVI	−23.174	0.34
	D	−63.506	0.25
	D + MVI	−19.346	0.66

*Abbreviations*: *UPDRS* Unified Parkinson’s Disease Rating Scale, *ADL* activities of daily living, *PDQ* Parkinson’s disease questionnaire, *SDMT* symbol digit modalities test, *LED* levodopa equivalent dose

^a^Model controlled for baseline age and the corresponding baseline score as fixed effects and including site as a random effect

## Discussion

In this analysis of the NET-PD LS1 cohort, we found that about 12% of subjects took > 400 IU/day of VitD supplementation while 34% took a MVI estimated to contain 400 IU/day of VitD. Disease progression as measured by a variety of rating scales at 3 years was similar in all 4 groups. Although methods of ascertainment were quite different, these rates of MVI and VitD use are in line with rates reported in the United States population from the large cross-sectional NHANES study of 34% for MVI use and 11% for separate VitD supplement use from 2007 to 8 [2].

Our finding that the age at diagnosis of PD was older in the MVI group, VitD and VitD plus MVI groups could suggest a disease delaying effect. However, further analysis of this data segregated according to age range of onset showed that VitD supplementation was higher in older participants (suggesting reverse causation), and that mean age at diagnosis was no longer significantly different between groups.

Our finding that VitD supplementation did not affect PD progression is consistent with observations from the DATATOP study of early PD in which no correlation was seen between VitD levels and disease progression [9]. However, a more recent 3-year study did find a small, but significant association between VitD levels measured at baseline and motor progression [10], raising the possibility that the amount of VitD supplementation by the NET-PD LS1 participants was inadequate to provide benefit.

Although there is evidence of high prevalence of VitD deficiency in early PD [9] and established PD [11], there is currently little published regarding the effect of VitD supplementation in PD. Suzuki et al. conducted a randomized, placebo-controlled study examining the effect supplementation with 1200 IU/day of vitamin D3/d for 12 months [12]. They found that D supplementation was associated with smaller changes in a number of outcomes measures including the Hoehn and Yahr stage, UPDRS part II and total, and some domains of the PDQ39 as well as the participants' VitD receptor genotype. Their study differed from our study in a number of ways since it was randomized, larger doses of VitD were specified, and because subjects started treatment after baseline while our study followed subjects on established VitD supplementation for a longer duration of time. Their design examines both potential treatment and neuroprotective effects of D supplementation while our study more specifically examined baseline characteristics of supplemented patients and their progression.

Our study has several limitations. As it is a secondary analysis, subjects were not randomized to treatment. Also, subjects were analyzed according to reported supplement dose with no independent measurements of serum VitD levels nor were we able to account for differing levels of sunlight exposure. Moreover although many subjects provided information regarding supplement use for years prior to study entry, study procedures only required report of supplement use in the 180 days prior to baseline, which for the majority of subjects was after their PD diagnosis. There were also lower numbers of subjects taking VitD or VitD+MVI compared to the other groups. Finally, while this study did not show differences in disease progression in the first three years after starting dopaminergic treatment, it is possible that D supplementation could affect later PD progression.

## Conclusion

Although VitD insufficiency is common in PD, this study shows VitD > 400 IU/day supplementation was not common and was similar to the rate observed in cross-sectional studies of the general US population according to age group. Although no difference in early disease progression was observed in patients taking VitD supplementation, it remains possible that consistent VitD supplementation could reduce the development of disability related to bone health, since osteoporosis is more common in PD and the risk of falls and bone fracture increase with disease duration. These data may be helpful in the design of studies testing the effect of vitamin D supplementation over a longer time frame.

## Abbreviations

ANOVA: Analysis of variance
LED: Levodopa equivalent dose
LS-1: Long-term study
MVI: Multivitamin
NET-PD: National Institutes of Health Exploratory Trials in Parkinson’s Disease
NoSupp: No supplement
PD: Parkinson’s disease
PDQ39: Parkinson’s Disease Questionnaire
SCOPA-COG: Scales for Outcomes in Parkinson’s Disease-Cognition
SDMT: Symbol Digit Modalities Test
UPDRS: Unified Parkinson’s Disease Rating Scale
Vitamin D: VitD
